# COVID-19 Pandemic and Personality: Agreeable People Are More Stressed by the Feeling of Missing

**DOI:** 10.3390/ijerph182010759

**Published:** 2021-10-13

**Authors:** Stephan Getzmann, Jan Digutsch, Thomas Kleinsorge

**Affiliations:** Leibniz Research Centre for Working Environment and Human Factors, Technical University of Dortmund (IfADo), Ardeystrasse 67, D-44139 Dortmund, Germany; digutsch@ifado.de (J.D.); kleinsorge@ifado.de (T.K.)

**Keywords:** COVID-19 pandemic, perceived stress, personality, longitudinal analysis

## Abstract

The COVID-19 pandemic and the measures taken to contain it have substantial consequences for many people, resulting in negative effects on individual well-being and mental health. In the current study, we examined whether individual changes in perceived stress relative to pre-pandemic levels depended on differences in behavior, appraisal, and experience of pandemic-related constraints. In addition, we tested whether this potential relationship was moderated by personality traits. We conducted an online survey during the end of the first lockdown in Germany in spring 2020, and assessed pandemic-related individual consequences as well as perceived stress. These data were related to the big five personality traits and to ratings of perceived stress obtained from the same participants in a study conducted before the outbreak of the pandemic, using the same standardized stress questionnaires. There was no overall increase, but a large interindividual variety in perceived stress relative to pre-pandemic levels. Increased stress was associated especially with strong feelings of missing. This relationship was moderated by agreeableness, with more agreeable people showing a higher association of the feeling of missing and the increase of perceived stress. In addition, openness and conscientiousness were positively correlated with an increase in stress. The results highlight the importance of considering personality and individual appraisals when examining the consequences of the COVID-19 pandemic on perceived stress and well-being.

## 1. Introduction

The COVID-19 pandemic has implications for many areas of life for people worldwide. It is not only the threat of infection with the corona virus and the disease itself and the possible loss of relatives and friends that play a role. In addition, the measures taken to contain the pandemic, which can be subsumed with the term “social distancing”, have a wide range of consequences for social life, such as leisure behavior, mobility and working life. For example, with the first lockdown ordered in Germany in spring 2020, the closure of pubs and stores, childcare facilities and schools, sports and cultural facilities, the restrictions on mobility and travel, and the increase in home office (where I am currently writing this introduction), there were significant restrictions on people’s everyday lives (e.g., [1]). In addition, many people faced financial concerns and feelings of insecurity and loss of control that many experienced as stressful, if not threatening. As a consequence of these rather indirect effects of the COVID-19 pandemic, studies reported a decrease in mental health in the general public, with higher rates of loneliness, anxiety, and depressive symptoms (e.g., [2,3,4]; for meta-analyses, [5,6]). In addition, lower psychological well-being was reported as compared to before COVID, with this decrease in well-being being associated with several factors, such as female gender, poor-self-related health and relatives infected with the corona virus [7]. In this context, the results of longitudinal studies examining mental health before and during the pandemic in the same population of individuals are particularly informative. For example, a British longitudinal study of mental health found an increase in mental distress compared with pre-COVID levels, with some groups of people showing a significantly greater increase in mental distress than others [8]. Another longitudinal study found no overall increase in clinically relevant mental health disorders such as major depression and generalized anxiety disorder relative to pre-COVID levels [9].

A common reaction to threatening situations, such as accidents and natural hazards, but also very personal events of emotional strain (such as illnesses or the loss of a job) is stress. In addition to the immediate stress reaction that occurs, for example, in response to an attack, longer-lasting life and work situations that are perceived as burdensome or threatening can lead to an increase in stress reaction and a habitually elevated stress level (e.g., [10,11]). In view of the consequences of the COVID-19 pandemic and the lockdown for various areas of life, an increase in stress levels can also be expected. However, previous research results on the COVID-19 pandemic provide a mixed picture on the relationship between the consequences of the pandemic and the increase in perceived stress. While some studies indicated a high prevalence of COVID-related stress (for meta-analyses, [12,13]), other did not found a consistent increase in stress levels (e.g., [14,15], and also no significant changes in perceived stress over the course of the pandemic [16]. However, a recent longitudinal study revealed that large interindividual variance in dealing with threatening and stressful events in general also seems to be evident in COVID-19 pandemic: While some individuals showed a significant increase in perceived stress, the majority of participants reacted rather calmly to the pandemic [17]. Results such as this suggest that a differentiated view of the consequences of the pandemic is necessary, also with regard to possible interventions to support particularly vulnerable groups.

Interindividual differences in perceived COVID-related stress may have a variety of causes. In addition to socio-demographic factors such as gender, age, socio-economic and health status (for review, [3,5]), differences in current attitudes and feelings, in the individual appraisal of the situation and the resulting consequences in behavior and experience can play a role (e.g., [18,19]). Coping strategies are also important here (e.g., [20,21,22]). Coping strategies in general can broadly be subdivided into either problem-focused coping (i.e., efforts to improve a given situation) or emotion-focused coping (i.e., efforts to regulate emotional distress) ([23], for review, [24]). For example, rather positive appraisal of the pandemic, social support, and adaptive cognitive emotion regulation were found to be positively, perceived stress and loneliness negatively associated with mental health [17]. Likewise, strategies such as active coping, using emotional support, and controllable-by-self appraisals were positively associated with positive affect. In contrast, threat appraisals, denial, and self-blame were positively associated with negative affect during the early phase of the pandemic [19].

In addition to these effects of appraisal and handling of stressful experiences, personality traits also seem to play a role. A relationship between coping with COVID-19 pandemic and personality has been demonstrated in some studies, such as with regard to the big five personality traits (e.g., [18,25,26]). In the big five framework, personality is conceptualized along five broad dimensions. These include “extraversion” (being positive, assertive, energetic, social, and talkative), “neuroticism” (being tense, anxious, hostile, impulsive, depressive, and of low self-esteem), “openness” (being curious, insightful, flexible, intellectual, and open for new impressions and experiences), “agreeableness” (being forgiving, kind, generous, trusting, sympathetic, compliant, and altruistic), and “conscientiousness” (being organized, efficient, reliable, self-disciplined, achievement-oriented, and rational) [27,28]. There is some empirical evidence that personality traits, appraisal/experience of a current situation, and the perceived stress are interrelated (e.g., [29,30]).

Regarding the COVID-19 pandemic, recent findings also indicate a relationship and interactions of personality traits, individual evaluations and perceptions, and the effects on stress experience [15,26,31,32]. Here, moderating effect of personality traits on appraisal and experience as well as coping with the COVID-19 pandemic have been observed. For example, a recent study found that the personality traits openness, extraversion, conscientiousness, and agreeableness were related to problem-focused coping (including seeking of social support), while neuroticism was related to maladaptive emotional coping of the consequences of COVID [33]. Such findings suggest that influences of appraisal and experience on COVID-related stress should not be considered in isolation, but in conjunction with longer-term (personality) traits. In particular, analysis of possible moderating effects of personality on the relationship of appraisal, experience, and stress could help explain why some people seem to suffer more from the pandemic than others.

One problem with most previous studies on COVID and stress is that there are usually no prior measurements of perceived stress. Asking participants to estimate the change in stress compared to a pre-COVID level makes the results susceptible to subjective bias. In some studies, before-after comparisons were made in two comparable cohorts, and post-COVID values were compared with values immediately before the outbreak of the pandemic (e.g., [25]). However, individual changes in perceived stress level compared to a pre-COVID level cannot be determined in this way. Another problem is that large-scale surveys often use non-standardized methods to assess stress, which makes the interpretation of the results difficult. However, there are some longitudinal studies using standardized measures, for example, a study by Voltmer et al. on students surveyed before and during the pandemic that showed no significant changes in perceived stress [34]. Finally, the determination of personality traits before the outbreak of the pandemic is also more favorable than a subsequent assessment, since findings show that these can deviate in their assessment compared to the pre-COVID assessment [35].

**Figure 1:**
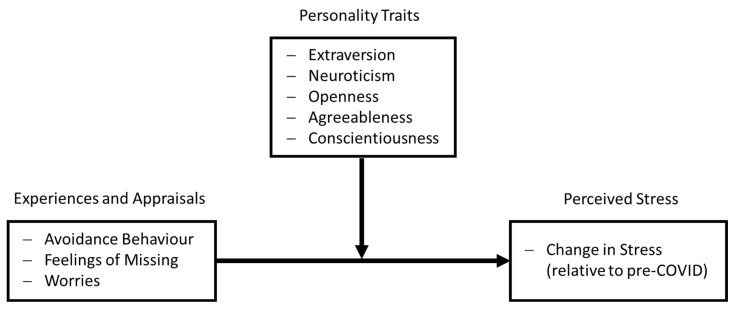
Schematic model of the assumed association between COVID-related experience and appraisals, personality traits, and changes in perceived stress.

The aim of the present study was to explore relationships between experiences and appraisals of the pandemic, personality traits, and resulting changes in perceived stress by means of standardized procedures on one and the same population of participants. To this end, participants of the Dortmund Vital Study, a broad-based study on determinants of healthy cognitive aging ongoing since 2016, were surveyed about the COVID-19 pandemic. First, personal experiences, appraisals, and behavior during the lockdown in spring 2020 in terms of avoiding potentially risky situations, the resulting feelings of missing, and the extent of worries in relation to the pandemic were queried. For instance, participants were asked about (a) the extent to which they avoided going to stores and meeting friends, (b) how much they missed doing so, and (c) how concerned they were about their health and that of their relatives. Second, perceived stress was determined using two well-established, standardized survey instruments. These parameters were related to stress data measured in the Dortmund Vital Study before the outbreak of the COVID-19 pandemic, and the relationships of experience and appraisal of the pandemic and changes in ratings of perceived stress were determined. Finally, potentially moderating effects of big five personality traits were determined (Figure 1).

## 2. Materials and Methods

### 2.1. Participants and Procedure

All participants in the present corona survey were registered subjects of the Dortmund Vital Study. This ongoing, large-scale study was launched in 2016 and investigates endogenous and exogenous factors influencing healthy aging in a sample of subjects aged 20 to 70 years. Participants of the Dortmund Vital Study had been recruited through social media appeals, newspaper advertisements, flyers, and promotions at public events and at companies in and near Dortmund, Germany. Since work-related factors are at the focus of the study, preference is given to employed persons. As part of the Dortmund Vital Study, the participants pass through several stations in which, in addition to the measurement of cognitive and physical performance, a variety of sociodemographic data, personality traits and stress perception are queried. The participants receive remuneration for their participation. The Dortmund Vital Study as well as the corona survey conformed to the Code of Ethics of the World Medical Association (Declaration of Helsinki) and was approved by the local Ethical Committee of the Leibniz Research Centre for Working Environment and Human Factors, Dortmund, Germany. All participants gave their written informed consent before any study protocol was commenced.

The corona survey was conducted as an online survey. For this purpose, all 580 registered participants of the Dortmund Vital Study who had taken part up to that point received an invitation to participate in the corona survey by e-mail. They were informed about the content and purpose of the survey and received a questionnaire on corona-specific topics as well as two standardized instruments for stress measurement. The invitation emails were sent out on 3 July 2020. This date was roughly after at the end of the first lockdown in Germany, which had been in effect since 22 March 2020 during which restrictions on public and private life were in place. These included the closure of stores, schools, restaurants, cultural and sports facilities, tourist accommodation and restrictions on freedom of movement. The first relaxations from lockdown for stores, popular and recreational outdoor sports, or visits to clinics started in 4 May, while contact restrictions extended until 5 June. The survey was completed mostly (88%) between 3 and 9 July, and (12%) between 10 and 29 July. By the end of the survey period on 31 July, *n* = 147 participants had responded. Of these, 139 had completely filled out the questionnaires on corona-specific aspects and on stress perception. These subjects were included in the analysis. The pre-COVID measurements of these subjects in the context of the Dortmund Vital Study took place in a period from 2016 April to March 2020. The average time between pre- and on-COVID measurements were 22.5 month, SD 13.2 months. The sample was 63.3% female and 36.7% male (“other” was not an option in this survey), 63.3% were employed, 10.8% were in education, 25.9% were not employed or retired. The mean age of the participants was 48.5 years (SD = 13.8; range: 20–70; age distribution: 20 to 29 years: 12.2%; 30 to 39 years: 12.3%; 40 to 49 years: 21.5%; 50 to 59 years: 29.5%; 60 to 70 years: 24.5%).

### 2.2. Measures

Stress measurement. Perceived stress was assessed using two standardized questionnaires, the German versions of the perceived stress questionnaire (PSQ; [36]) and the Trier Inventory for Chronic Stress (TICS; [37]). The employed short version of the PSQ (PSQ20; [38]) consists of 20 items assessing various stress-related factors (worries, tension, joy, demands). Participants rated on a 4-point scale how often they had experienced certain feelings in the last four weeks (1 = almost never, 2 = sometimes, 3 = often, 4 = most of the time). The TICS consists of 57 items assessing nine interrelated factors of chronic stress (work overload, social overload, pressure to perform, work discontent, work demands, lack of social recognition, social tensions, social isolation, and chronic worries). Participants rated how often they have experienced certain situations or have had certain experiences within the last three months on a 5-point scale (0 = never, 1 = rarely, 2 = sometimes, 3 = often, 4 = very often). Total scores were calculated over all items of PSQ20 and TICS.

Big five personality. Personality was assessed using the German version of the NEO-FFI Personality Inventory [28] consisting of 60 items, in which self-reports of five personality dimensions (neuroticism, extraversion, openness, agreeableness, and conscientiousness) were rated on a 5-point scale (1 = strong disagreement, 2 = disagreement, 3 = neutral, 4 = agreement, 5 = strong agreement).

COVID-related questionnaire. In order to assess the participants’ behavior, attitudes and feelings regarding the COVID-19 pandemic, a questionnaire was designed. This questionnaire covered the aspects of mobility, changes in leisure time behavior/leisure time activities and daily life, personal appraisals and opinions, health, media and information, and more general information. For the present study, the focus was set on three different aspects:

(a) Avoidance behavior: “are there activities you consciously avoid to reduce the risk of corona infection? I avoid (1 = completely, 2 = somewhat, 3 = hardly, 4 = not at all):
  − visits of stores
  − use of bus and train
  − visits of close relatives
  − visits of close acquaintances/friends
  − staying in public places
  − spatial proximity to people on the street
  − leaving one’s own home”;
(b) feelings of missing: “To what extent do you miss the following activities due to the existence of the corona crisis limitations? I miss (1 = very much, 2 = somewhat, 3 = not at all):
  − visiting acquaintances/friends
  − going dancing
  − visiting restaurants, clubs, bars
  − visit cultural events, e.g., theater, concerts, museums, festivals
  − hiking/walking
  − fitness/sports
  − go to private parties
  − go on excursions
  − go out for dinner
  − spending time with family members outside the home
  − attend educational events
  − go shopping
  − go on vacation”;
(c) worries: “In the light of the COVID-19 pandemic, are you personally concerned about the following areas of your life? I am concerned about (1 = very much, 2 = very, 3 = somewhat, 4 = little, 5 = not at all):
  − my physical health
  − my mental well-being
  − the health of my relatives/close acquaintances
  − economy and prosperity
  − school and education system
  − care and health system in Germany
  − social peace in Germany
  − solidarity in the close environment
  − world peace”.

For reliability analysis, Cronbach’s alpha was calculated for each scale, indicating satisfying internal consistency for avoidance behavior, 0.807, feelings of missing, 0.820, and worries, 0.829. In order to obtain a total value per category, mean rating scores were averaged for each subject and then z-transformed.

### 2.3. Data Analyses

As a first step of analysis, pre-COVID ratings of perceived stress were compared to on-COVID ratings. Mean values of the PSQ and the TICS were calculated for each subject, by averaging the scores of the four subscales of the PSQ and the nine subscales of the TICS. The averaging was performed in a way that higher scores indicated higher perceived stress ratings. For mean values and for each subscale of PSQ and TICS, pre-/on-COVID ratings were compared using student t-tests. All p-values were corrected for false discovery rate (FDR; [39]). Then pre-/on-COVID difference values were calculated by subtracting the pre-COVID mean values from the on-COVID mean values, separately for PSQ und TICS. Thus, positive difference values represent an increase in perceived stress, while negative values represent a decrease in stress. Finally, a single and comprehensive difference measure was determined for the further analyses, termed “Δstress”: the pre-/on-COVID difference values of PSQ and TICS were therefore z-transformed and then aggregated. The idea behind this aggregation across both stress measures into a single measure Δstress was that changes in perceived stress could be based on a larger number of observations and that the number of tests for statistical significance (and therefore the risk of Type-1 errors) could be reduced.

As the second part, a correlational analysis was employed to test whether the COVID-related appraisals and feelings, the big five personality traits, and the change in perceived stress were interrelated. Pearson-correlation coefficients of the scores of “avoidance behavior”, “feelings of missing”, and “worries”, personality traits, and Δstress were calculated. Third, for regression analysis, we used the PROCESS 3.5 macro for SPSS (Regression Model Number 1; [40]) to test whether correlations of COVID-related appraisals and feelings (X) and changes in perceived stress levels (Y) were moderated by personality traits (M). PROCESS uses ordinary least squares regression and yields unstandardized coefficients for all effects. Confidence intervals were determined using bootstrapping (5000 samples) together with heteroscedasticity consistent standard errors (HC3; [41]).

Finally, in order to test whether participants in the Dortmund Vital Study who took part in the corona survey differed in age, gender, personality, or pre-COVID ratings of perceived stress from those who did not, between-subjects *t*-tests were calculated.

## 3. Results

### 3.1. Changes in Chronic Stress Level

The pre-COVID vs. on-COVID comparisons of perceived stress levels did not indicate significant differences in the overall test scores, neither for the PSQ, nor the TICS (Table 1). However, the analysis of specific subscales of stress revealed some significant differences: PSQ subscales “joy” decreased, and “social isolation” increased, while work-related subscales of the TICS indicated a decrease in “work overload” and “pressure to perform”, however an increase in “work discontent”.

**Figure 2:**
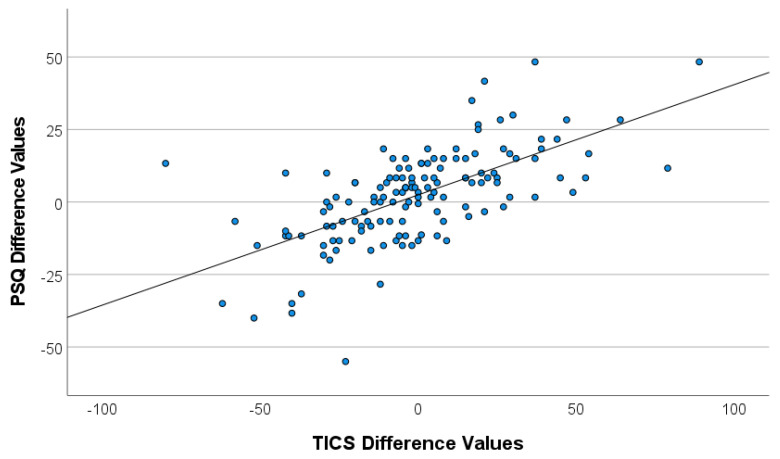
Scatterplot of pre-/on-COVID difference values of PSQ and TICS.

Both PSQ and TICS indicated a great interindividual variability in the overall pre-/on-COVID difference scores (Figure 2), suggesting profound differences in changes of perceived stress between participants. In other words, while some participants reported a significant increase in perceived stress (difference values > 0), others reported perceiving less stress relative to the pre-COVID levels (difference values < 0). A significant correlation occurred between the (z-transformed) pre-post differences scores of PSQ and TICS, *r* = 0.64; *p* < 0.001. For further analyses on changes of perceived stress, a single difference value (Δstress) was determined (see Section 2.3). There was no effect of gender on Δstress (male: mean Δstress 0.07, SD 0.68; female: mean Δstress −0.04, SD 1.02; *t*_137_ = 0.715, *p* = 0.476), while a slight, but significant negative correlation of age and Δstress, *r* = −0.17; *p* = 0.047, indicated that higher age was associated with lower increases in perceived stress.

### 3.2. Relationships of COVID-Related Appraisals and Feelings on Δstress

**Figure 3:**
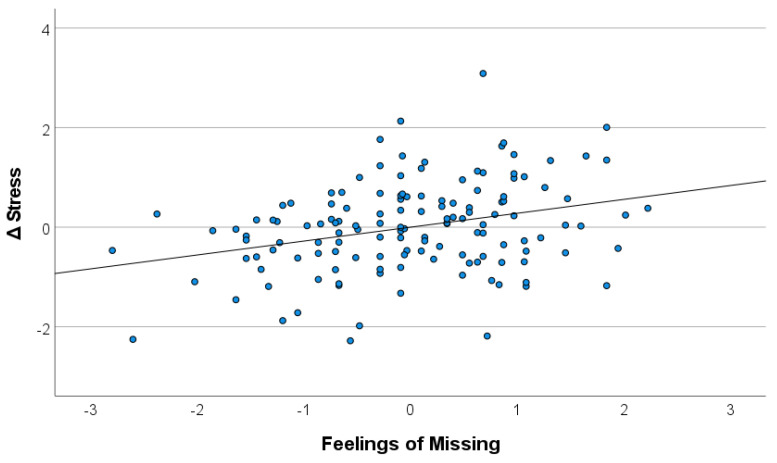
Scatterplot of z-transformed pre-/on-COVID difference values (Δstress) and ratings of “feelings of missing”.

There was a highly significant correlation between the ratings of “feelings of missing” and Δstress, *r* = 0.308; *p* < 0.001 (Table 2), indicating that participants with stronger feelings of missing experience a higher increase in perceived stress relative to pre-COVID levels (Figure 3). Ratings of “avoidance behavior” were not associated with Δstress, while there was a small, but significant correlation between Δstress and ratings in “worries”, *r* = 0.190; *p* = 0.025. Higher ratings in “feelings of missing” were associated with higher ratings in “avoidance behavior”, *r* = 0.275; *p* < 0.001, and “worries”, *r* = 0.483; *p* < 0.001, while “avoidance behavior” and “worries” were not correlated. The relationship between “feelings of missing” and Δstress remained stable even when controlled for effects of “avoidance behavior” and “worries”, according to a significant partial correlation, *r* = 0.270; *p* = 0.001.

### 3.3. Relationships of COVID-Related Appraisals and Feelings and Personality

There were no correlations between personality traits and COVID-related appraisals and feelings (Table 2), except from a highly significant positive correlation of neuroticism and “worries”, *r* = 0.259; *p* < 0.001: Individuals scoring higher on the neuroticism scale worried more about COVID-related issues.

### 3.4. Effects of Personality Traits and Moderating Effects of Personality Traits on Relationship of Δstress and “Feelings of Missing”

There were significant correlations of Δstress and personality traits openness, *r* = 0.187; *p* = 0.027, conscientiousness, *r* = 0.196; *p* = 0.021, and agreeableness, *r* = 0.191, *p* = 0.024, indicating that individuals scoring higher on these personality scales reported a higher increase in perceived stress compared to pre-COVID level. No relationships were found for extraversion or neuroticism (Table 2).

**Figure 4:**
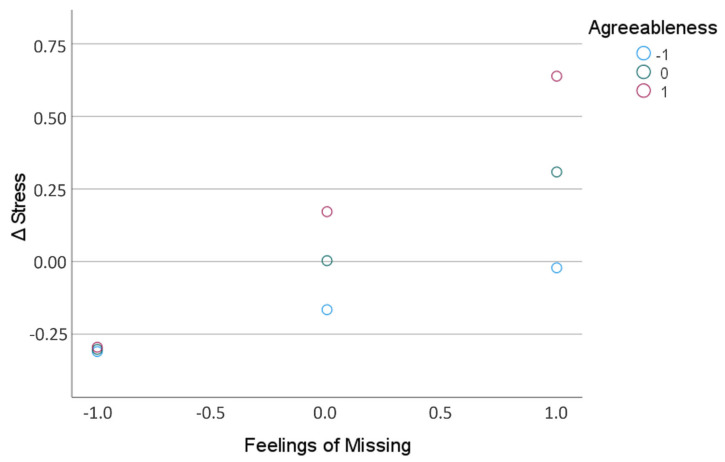
Changes in perceived stress (Δstress) as function of “feelings of missing” for persons scoring relatively high, medium, and low on the big five agreeableness scale.

Finally, we tested whether the observed relationship of “feelings of missing” (X) and Δstress (Y) was moderated by personality traits (M), using regression model number 1 of the PROCESS 3.5 macro for SPSS (Hayes, 2018). There were no moderating effects for extraversion, F_1,135_ = 0.858; *p* = 0.356, 95% CI[−0.07, 0.19], neuroticism, F_1,135_ = 3.200; *p* = 0.076, 95% CI[−0.20, 0.03], openness, F_1,135_ = 1.274; *p* = 0.261, 95% CI[−0.25, 0.07], or conscientiousness, F_1,135_ = 0.755; *p* = 0.386, 95% CI[−0.08, 0.21]. However, agreeableness significantly moderated the effect between “feelings of missing” and Δstress, F_1,135_ = 7.204; *p* = 0.008, 95% CI[0.05, 0.28]. Thus, when reporting strong “feelings of missing”, agreeable persons showed the most pronounced increase in perceived stress, while persons scoring low on the agreeableness scale showed rather no changes in stress. When reporting only weak “feelings of missing”, persons showed rather a decrease in perceived stress relative to pre-COVID level, irrespective of whether they score low or high on the agreeableness scale (Figure 4).

### 3.5. Comparison of the Participating and Non-Participating Subjects

The comparison of the participants of the Dortmund Vital Study who took part in the corona survey and those who did not revealed that participants in the survey were older than nonparticipants (48.5 vs. 43.1 years; *t*_578_ = 3.92; *p* < 0.001). However, the two groups did not significantly differ in any of the variables examined (stress according to Trier Inventory for Chronic Stress, 72.27 vs. 74.97; *t*_577_ = 0.90; *p* = 0.371; stress according to the perceived stress questionnaire, 32.43 vs. 35.68; *t*_577_ = 1.75; *p* = 0.080; extraversion, 27.94 vs. 29.06; *t*_578_ = 1.77; *p* = 0.077; neuroticism, 18.10 vs. 18.57; *t*_578_ = 0.62; *p* = 0.537; openness, 31.14 vs. 30.36; *t*_578_ = 1.23; *p* = 0.218; agreeableness, 32.02 vs. 32.75; *t*_578_ = 1.27; *p* = 0.207; and conscientiousness, 34.06 vs. 34.44; *t*_578_ = 0.63; *p* = 0.531). Neither there were significant differences in gender distribution (63.3/36.7 vs. 61.0/39.0; Chi^2^ = 0.239; *p* = 0.625).

## 4. Discussion

Overall, no consistent increase in perceived stress occurred relative to pre-pandemic levels; neither PSQ nor TICS showed significant change from pre-COVID measurements. However, large interindividual differences between participants did occur: while some participants reported a large increase in the level of perceived stress, most respondents showed little change, and some respondents even reported a decrease in perceived stress. This finding is consistent with results from previous studies (e.g., [14,15,16]). A recent longitudinal study that surveyed students in Germany before and during the pandemic also found no changes in perceived stress overall, but differences among participants [34]. In particular, a group of more vulnerable students could be identified. Some of these students have reported having mental health problems (like anxiety or depression) already before the pandemic. A further longitudinal analysis also revealed high interindividual variance in changes in stress levels over the course of the pandemic [17]. Here, the authors identified three groups of participants, one of which was a vulnerable group that showed a substantial increase in stress level over the course of the lockdown, while other groups tended to show a decrease in stress level. These and other findings highlight the need for a differentiated analysis of the interaction of various influencing factors on changes in stress levels.

Important influencing factors (besides socio-demographic factors such as gender, age, social environment and personal concern, see [5], for review) are also the individual handling of the pandemic-related consequences of the lockdown and their appraisals (e.g., [6,18,19,31]). The focus in this paper was on three aspects of behavior and evaluation. These were (a) the extent to which respondents are worried (for example, about their health or the impact of the corona-related measures of the lockdown), (b) the active avoidance of potentially dangerous activities during the lockdown (such as using public transport or avoiding crowds), and (c) the associated feelings of missing (for example, meeting friends or visiting restaurants). The analysis of the relationships between these three aspects revealed significant positive correlations, according to which a high level of worrying was associated with more pronounced avoidance behavior and consequently stronger feelings of missing. In addition, an increase in perceived stress compared to the pre-COVID level was mainly associated with feelings of missing, to a lesser extent with the extent of worrying, but not with avoidance behavior per se. Overall, it seems plausible that individuals who worried a lot and had a strong feeling of missing something reported a higher increase in perceived stress than those who did not worry and did not miss anything. In line with this observation, a daily dairy study on the effects of worry and affect on perceived stress suggested that mobilization of positive emotion could be a way to reduce COVID-related stress [42].

The relationships between changes in perceived stress and personality traits were rather weak: individuals with high scores on openness, agreeableness, and conscientiousness showed a higher increase in perceived stress, although the relationships were not particularly strong. In contrast to several previous studies (e.g., [18,32]; over overview, [43]), extraversion and neuroticism did not seem to play a role here. It is also noticeable that agreeableness was associated with lower levels of stress, anxiety, and depressive symptoms in previous studies [26,44,45], whereas in the present analysis a positive correlation of agreeableness and increase in perceived stress was observed. One possible reason for this discrepancy could be that in the present study not absolute stress ratings at the time of the survey were analyzed, but the change in stress ratings relative to a time point before the outbreak of the pandemic. However, also at a longitudinal level, associations with personality traits have been observed, such as a greater increase in perceived stress among less extraverted students [15]. The question, though, is to what extent this population is comparable to the one in the present study.

Furthermore, low were the correlations between COVID-related scores and feelings and personality variables: here, neurotic individuals were found to worry more than less neurotic individuals. This observation is in line with previous results (e.g., [45]) and seems very plausible in view of the characterization of neuroticism as the tendency to experience anxiety, tension, and irrational thinking [27,28]. Remarkably, these individuals did not show higher levels of avoidant behavior than less neurotic participants. Given that extraversion is characterized by a high need for social interaction, it is also striking that high scores on the extraversion scale were not associated with greater feelings of missing. However, this observation is consistent with previous results showing that extraverted individuals did not per se suffer more from social distancing than less extraverted individuals [46]. A possible reason could be that extraverts managed quite well to maintain or regain their social connectedness during the lockdown. Finally, the regression analysis revealed that the relationship between the strength of the feeling of missing and the change in perceived stress was moderated by the personality trait agreeableness. In this context, a strong increase in perceived stress occurred primarily in agreeable people who reported strong feelings of missing.

Agreeable people are characterized by personality traits characterized as kind, generous, trusting, sympathetic, compliant, altruistic, and trustworthy [27,28] and are described as peaceful, helpful [47]), and empathic [48]. They exhibit a higher degree of resilience than less agreeable people (according to a meta-analysis, [49]) and show an overall desire to maintain positive relationships [50], most of which result in strong social networks [51]. Previous findings on the associations between personality and stress suggested differences in the use of coping strategies that may also play a role here. For example, high scores on the neuroticism scale appear to be more associated with negative (maladaptive) emotion-focused strategies, such as escape avoidance, hostile reactions, and emotional venting. In contrast, high scores on the extraversion scale appear to be more associated with problem-focused strategies, such as planning and rational action (e.g., [52]). A quite similar pattern was recently described in the context of the COVID-19 pandemic [33]. Regarding agreeableness, fewer studies are available, but they suggest that agreeableness is more likely to be associated with the use of problem-focused and positive emotion-focused strategies (for a meta-analysis, [53]). Thus, associations are described between high levels of agreeableness and strategies such as seeking social support [51,54], passive endurance [52], avoiding confrontation [55] as well as conflict resolution strategies [50].

In view of the current findings, it could therefore be that individuals with high agreeableness scores were unable to use their preferred coping strategy of social support seeking, or only to a reduced extent, due to the contact restrictions. In this context, the feelings of missing could have played a double role, namely (a) related to the not possible social activities per se, and (b) related to the thus omitted possibility of coping through these social activities. In other words, agreeable people who missed social activities as a result of the lockdown were unable to alleviate the resulting stress through social interaction with other people, resulting in high values in the increase of perceived stress. Those (agreeable) persons who missed little had lower overall levels of stress to cope with and therefore perceived comparatively little stress. In this context, the results of a study on the effects of the COVID-19 pandemic and personality on well-being are interesting, showing that the effect of agreeableness on positive affect was reduced by the pandemic [25]. Again, this could be due to the lack of social interactions and the positive affect that could result. Conversely, less agreeable individuals who rely to a lesser degree on social support might had far less stressful experience: even at high levels of feelings of missing, they could more easily adapt to the measures of social distancing [56], using more “socially-independent” coping strategies. In this context, it is worth mentioning that in the present analysis agreeable people showed not per se stronger feelings of missing and that the moderating effect of personality found was not solely due to a higher sensitivity of agreeable people to the consequences of the lockdown.

An alternative interpretation of the moderating role of agreeableness on the relation between the feeling of missing and increased stress could be that agreeable people tend to renounce their own needs more than less agreeable people. In other words, less agreeable people may try to resort to alternative ways of need fulfillment which elude social control despite being forbidden by governmental measures (e.g., meeting with friend at home instead of in public places). In line with this assumption, cross-sectional studies revealed that lower agreeableness scores were associated with less acceptance of COVID-19 containment measures [45], and that less agreeable people are less likely to follow the rules of, for example, social distancing [57]. However, it should be noted that there was no association between agreeableness and avoidant behavior in the present analysis, suggesting that agreeable people did not adhere to the corona-related measures of contact reduction and social distancing more than less agreeable people (or at least the latter did not admit it).

An interesting secondary finding of the study is the change in perceived stress in specific subcategories of stress experience. First, stress ratings in PSQ subscales “joy” and “social isolation” differed relative to pre-COVID values. These findings of reduced enjoyment of life and increased social isolation are frequently described and might be viewed as direct consequences of the measures taken to contain the spread of corona virus (for meta-analyses, [5,6]. Second, the TICS revealed a decrease in perceived “work overload” and “pressure to perform”, but also an increase in “work discontent”. It must be mentioned in this context that the participants are part of the ongoing Dortmund Vital Study, which is closely related to the world of work, and for which working people are preferably recruited. Accordingly, about 75% of the respondents are employed or in education. An additional analysis indicated that about 53% of this group stated that they work entirely or at least partly from home. This proportion roughly corresponds to the proportion of people who worked from home in Germany during the first lockdown (61%; [58]). It is therefore possible that the workload and the pressure to perform decreased during the pandemic as a result of working in a home office or reduced contact with colleagues at the workplace, but also as a result of measures such as short-time working. What is remarkable, however, is that job satisfaction seems to have decreased at the same time.

Comparable evidence was found in studies on the effects of telework on employees during the COVID pandemic (e.g., [59]). For example, a study conducted in Germany during the first lockdown also found less stress and higher life satisfaction among home office employees compared to those who did not work at home [60]. Feelings of greater security in the home environment (and thus a lower fear of infection with the corona virus), more autonomy and flexibility, and also the absence of commuting were cited as possible reasons. The authors interpret this within the framework of the theory of conservation of resources [61]. According to this theory, the reduction of these burdens may have freed up additional resources, which could have led to a decrease in perceived workload and performance pressure. Interestingly, this study also found a reduction in perceived control over work and reduced feelings of control over career when working from home. This might be related to the reduced job satisfaction found in the present analysis. It should be noted, however, that the study by [60] examined the influence of telework during lockdown cross-sectionally. A comparison of these results with the longitudinal changes in subcategories of perceived stress might therefore be difficult. In general, the picture is rather mixed regarding the impact of telework on mental health (for review, [62]).

A strength of our analysis is certainly that the same participants could be examined by using the same measures of perceived stress before and during the pandemic. However, several limitations of our study should also be mentioned: first, self-selection cannot be ruled out due to the comparatively low response rate of 23.6% of the persons contacted. The comparison of the group of subjects who entered the analysis and the group who did not respond or responded incompletely indicated that the participating group was significantly older than the latter one. Thus, although observed also in previous studies (for meta-analyses, [6,12]), the finding that higher age was associated with a lower increase in perceived stress should be treated with caution. On the other hand, the analysis did show that there were no significant differences in personality and perceived stress measured before the pandemic between the two groups of subjects. Nevertheless, it is not clear to what extent the moderating effect of agreeableness found on the relationship between the feelings of missing and stress increase can be generalized. This is also true in view of the fact that the invited subjects were all participants in the Dortmund Vital Study, a large-scale study on the determinants of healthy aging. Although care was taken to ensure a high representativeness of the subject population (e.g., with regard to age distribution, education, and occupation), self-selection effects cannot be completely ruled out here either. A second issue is the focus on three specific aspects of the consequences of the COVID-19 pandemic, which in turn could be moderated by influencing factors (such as the social status of the respondents or their social environment). However, adding a larger number of potentially relevant variables did not seem appropriate given the comparatively small number of data sets. Finally, the timing of the survey might be critically questioned, which tended to coincide with the end of the first corona-related lockdown in Germany. At that time the most serious measures (such as curfews) had already been relaxed or completely cancelled. However, it should be noted that both survey instruments for perceived stress refer to a longer period, covering four weeks (PSQ) and three months (TICS), so that the period of active lockdown should be well covered.

## 5. Conclusions

The study demonstrates high inter-individual variance in COVID-related changes in perceived stress due to differences in appraisals and feelings as well as moderating influences of personality traits. Further longitudinal studies of associated coping strategies are desirable and needed to capture longer-term effects of the pandemic on perceived stress. The results also demonstrate the need to uncover moderating influences (for example, personality) on COVID-related impacts, and to identify patterns that characterize particularly vulnerable groups of people. Understanding these patterns better will make it possible to offer specific preventive measures to these people to prevent them from mental distress in future, similar situations.

## Figures and Tables

**Table 1:** Means (M) and standard deviations (SD) of PSQ and TICS stress subscales for pre-COVID vs. on-COVID measurements and results of paired *t*-tests (*n* = 139) with Cohen’s *d* as effect sizes. All *p*-values are corrected for false discovery rate (FDR; [39]), significant differences are printed in bold.

		Pre-COVID		On-COVID				
		M	SD	M	SD	*t*	*p*	*d*
PSQ	Worries	24.07	21.53	25.85	21.36	−1.06	0.395	−0.090
	Tension	33.69	22.76	36.31	26.00	−1.51	0.222	−0.128
	Joy	**63.80**	**22.83**	**56.12**	**24.64**	**4.76**	**>0.001**	**0.404**
	Demands	35.72	21.65	32.28	23.15	1.77	0.169	0.150
	Mean	32.43	19.00	34.58	19.92	−1.58	0.218	−0.134
TICS	Work Overload	**11.45**	**6.25**	**9.36**	**6.79**	**4.130**	**>0.001**	**0.350**
	Social Overload	9.66	5.41	8.83	5.50	2.175	0.078	0.184
	Pressure to Perform	**14.71**	**6.91**	**12.65**	**7.28**	**4.287**	**>0.001**	**0.364**
	Work Discontent	**8.98**	**5.26**	**10.23**	**6.28**	**−2.902**	**0.012**	**−0.246**
	Work Demands	5.42	3.92	5.75	4.41	−1.096	0.413	−0.093
	Lack of Social Recognition	4.99	3.35	4.96	3.55	0.137	0.891	0.012
	Social Tensions	5.96	4.45	5.80	4.48	0.547	0.675	0.046
	Social Isolation	**5.92**	**4.52**	**8.55**	**5.52**	**−5.941**	**>0.001**	**−0.504**
	Chronic Worries	5.18	3.70	5.34	3.68	−0.551	0.728	−0.047
	Sum	72.27	31.10	71.48	35.76	0.346	0.782	0.029

**Table 2:** Pearson-correlation coefficients of changes in perceived stress (Δstress), “avoidance behavior”, “feelings of missing”, “worries”, as well as big five personality traits, significant correlations are printed in bold.

	ΔStress	Avoidance Behavior	Feelings of Missing	Worries
Avoidance Behavior	−0.019			
Feelings of Missing	0.308 **	0.275 **		
Worries	0.190 *	0.204 *	0.483 **	
Extraversion	0.161	0.070	0.110	0.120
Neuroticism	−0.108	0.130	0.153	0.295 **
Openness	0.187 *	0.037	0.077	0.081
Agreeableness	0.191 *	0.020	−0.021	−0.014
Conscientiousness	0.196 *	0.074	0.110	0.025

* *p* < 0.05; ** *p* < 0.01.

## Data Availability

The data presented in this study are available on request from the corresponding author. The data are not publicly available due to ethical reasons.
